# Extracellular proteolytic activation of *Pseudomonas aeruginosa* aminopeptidase (PaAP) and insight into the role of its non-catalytic N-terminal domain

**DOI:** 10.1371/journal.pone.0252970

**Published:** 2021-06-16

**Authors:** Itschak Axelrad, Mary Safrin, Rivka Cahan, Sang-Jin Suh, Dennis E. Ohman, Efrat Kessler

**Affiliations:** 1 Maurice and Gabriela Goldschleger Eye Research Institute, Tel Aviv University Sackler Faculty of Medicine, Sheba Medical Center, Tel-Hashomer, Ramat Gan, Israel; 2 Department of Biomedical Sciences, Texas A&M College of Dentistry, Dallas, Texas, United States of America; 3 Department of Microbiology and Immunology, Virginia Commonwealth University Medical Center, Richmond, Virginia, United States of America; 4 McGuire Veterans Affairs Medical Center, Richmond, Virginia, United States of America; University of Sao Paulo, BRAZIL

## Abstract

*Pseudomonas aeruginosa* secretes several endopeptidases, including elastase, alkaline proteinase (Apr), a lysine-specific endopeptidase (LysC), and an aminopeptidase (PaAP), all of which are important virulence factors. Activation of the endopeptidases requires removal of an inhibitory N-terminal propeptide. Activation of pro-PaAP, in contrast, requires C-terminal processing. The activating proteases of pro-PaAP and their cleavage site(s) have not yet been defined. Studying pro-PaAP processing in a wild type *P*. *aeruginosa* strain and strains lacking either elastase or both elastase and Apr, we detected three processing variants, each ~56 kDa in size (AP56). Activity assays and N- and C-terminal sequence analyses of these variants pointed at LysC as the principal activating protease, cleaving a Lys_512_-Ala_513_ peptide bond at the C-terminal end of pro-PaAP. Elastase and/or Apr are required for activation of LysC, suggesting both are indirectly involved in activation of PaAP. To shed light on the function(s) of the N-terminal domain of AP56, we purified recombinant AP56 and generated from it the 28 kDa catalytic domain (AP28). The kinetic constants (*K*_*m*_ and *K*_*cat*_) for hydrolysis of Leu-, Lys-, Arg- and Met-p-nitroanilide (pNA) derivatives by AP56 and AP28 were then determined. The catalytic coefficients *(K*_cat_/*K*_m_) for hydrolysis of all four substrates by AP28 and AP56 were comparable, indicating that the non-catalytic domain is not involved in hydrolysis of small substrates. It may, however, regulate hydrolysis of natural peptides/proteins. Lys-pNA was hydrolyzed 2 to 3-fold more rapidly than Leu-pNA and ~8-fold faster than Arg- or Met-pNA, indicating that Lys-pNA was the preferred substrate.

## Introduction

*Pseudomonas aeruginosa* is an opportunistic pathogen that causes acute infections in immunocompromised and burn patients and in corneal infections of traumatized eyes [1,2]. It also produces chronic lung infections in cystic fibrosis patients and is associated with high morbidity and mortality [3,4]. The success of *P*. *aeruginosa* as a pathogen is attributed to the large number of virulence factors it produces, among them several secreted proteases that can cause substantial tissue damage, promote bacterial proliferation and invasion and interfere with host defense mechanisms [5–7].

At least eight secreted proteases of *P*. *aeruginosa* are currently known [2,7,8], including the endopeptidases elastase (also called pseudolysin or LasB, a term based on the name of the elastase coding gene, *lasB*; Ela is an acronym used here for elastase), alkaline proteinase (also called aeruginolysin; designated here Apr), staphylolytic protease (also called staphylolysin or LasA protease, a term derived from the name of the staphylolysin coding gene, *lasA*) and lysyl endopeptidase (also known as protease IV or PrpL, the latter is identical to the name of the respective gene; designated here LysC) and an aminopeptidase that releases single amino acids from peptides and proteins (also known as PaAP, an abbreviation of *P*. *aeruginosa* aminopeptidase, PA-LAP, an abbreviation of *P*. *aeruginosa* leucine aminopeptidase, and PepB, which is identical to the name of the aminopeptidase coding gene, *pepB*) [9,10].

Ela is the most abundant and potent protease of *P*. *aeruginosa* [11]. It has a broad cleavage specificity although it favors hydrophobic or aromatic amino acid residues, with preference to Phe and Leu at the P1′ position, i.e., cleavage occurs on the amino side of these residues [12]. The proteolytic activity of Apr is limited and its cleavage specificity remains unclear although it has been reported to cleave peptide bonds on the carboxyl side of arginine residues [13]. LasA protease cleaves exclusively peptide bonds after Gly-Gly pairs that are abundant in the cell wall peptidoglycan of *Staphylococci* and present in elastin. This unique specificity accounts for its ability to lyse *Staphylococci* and nick elastin [14,15]. LysC is a lysine specific serine protease that cleaves peptide bonds on the carboxyl side of lysine residues exclusively and is highly sensitive to the lysine-specific serine protease inhibitor Tosyl-lysyl-chloromethylketone (TLCK) [16,17].

PaAP is a heat-stable enzyme, reported to preferentially cleave N-terminal leucine residues [9]. It is encoded by *pepB* (chromosome no. PA2939 in the *P*. *aeruginosa* PAO1 chromosomal map; see Pseudomonas Genome Data base) and produced as a 536 amino acid residue pre-proenzyme encompassing a 24-residue signal peptide, a 248 residue thermo-sensitive N-terminal domain of unknown function, and a C-terminal heat-stable catalytic domain of 264 residues. The non-catalytic N-terminal domain contains a protease-associated (PA) domain that is believed to be involved in protein-protein interactions and/or substrate recognition [18–21]. PaAP is closely related to other bacterial aminopeptidases, in particular those of *Streptomyces griseus*, *Vibrio proteolyticus* and *Aeromonas caviae*, all belonging to the M28 family of Zn-metallopeptidases [9]. The catalytic domain (~28 kDa in size; designated AP28) can be released from the active form of PaAP (~56 kDa) by heating in the presence of Ela [9]. A heat stable aminopeptidase from *P*. *aeruginosa* that preferentially cleaves N-terminal lysine [22], a solvent-stable aminopeptidase from *P*. *aeruginosa* [23] as well as an aminopeptidase that can cleave a synthetic insecticide called β-Cypermethrin [24] have been described and are most probably identical to PaAP because they all share the same primary structure.

PaAP is secreted via the type II secretion pathway of *P*. *aeruginosa* [25,26] and is regulated transcriptionally by quorum-sensing [27] and the alternative Sigma factor RpoS [28,29]. PaAP was first identified as a 58 kDa putative aminopeptidase in a *P*. *aeruginosa* strain lacking Ela and Apr but not in a wild type or a mutant strain lacking Ela alone, in which, it appeared as a 56 kDa protein [26]. The identity of the 56 kDa protein as an aminopeptidase (designated AP56) was confirmed somewhat later by Cahan et al [9] who showed that it possesses aminopeptidase activity. N-terminal sequencing of AP56 suggested that activation involved proteolytic removal of the first 12–14 residues from AP58 [9]. Subsequent studies [30] revealed however that activation requires proteolytic removal of a short C-terminal sequence from the pro-enzyme, a conclusion inferred from *in vitro* studies on processing of recombinant pro-PaAP by Ela and several non-relevant proteases. In the current study, we extended their work to identify the protease(s) involved in maturation of PaAP in *P*. *aeruginosa* and define the relevant cleavage site(s). We confirmed that activation requires C-terminal processing and identified LysC as the principal activator. Ela and Apr may also be involved, apparently indirectly via activation of LysC. Purification of AP56 and AP28 (the catalytic domain) enabled comparison of the kinetic constants for hydrolysis of certain amino acid p-nitroanilide (pNA) derivatives by both forms of the enzyme. The results revealed that Lys-pNA is cleaved more efficiently than Leu-pNA and indicated that the non-catalytic domain in AP56 has little or no effect on hydrolysis of small synthetic substrates.

## Results

### Elastase (Ela) is sufficient but not essential for activation of Pro-PaAP (AP58)

**Fig 1:**
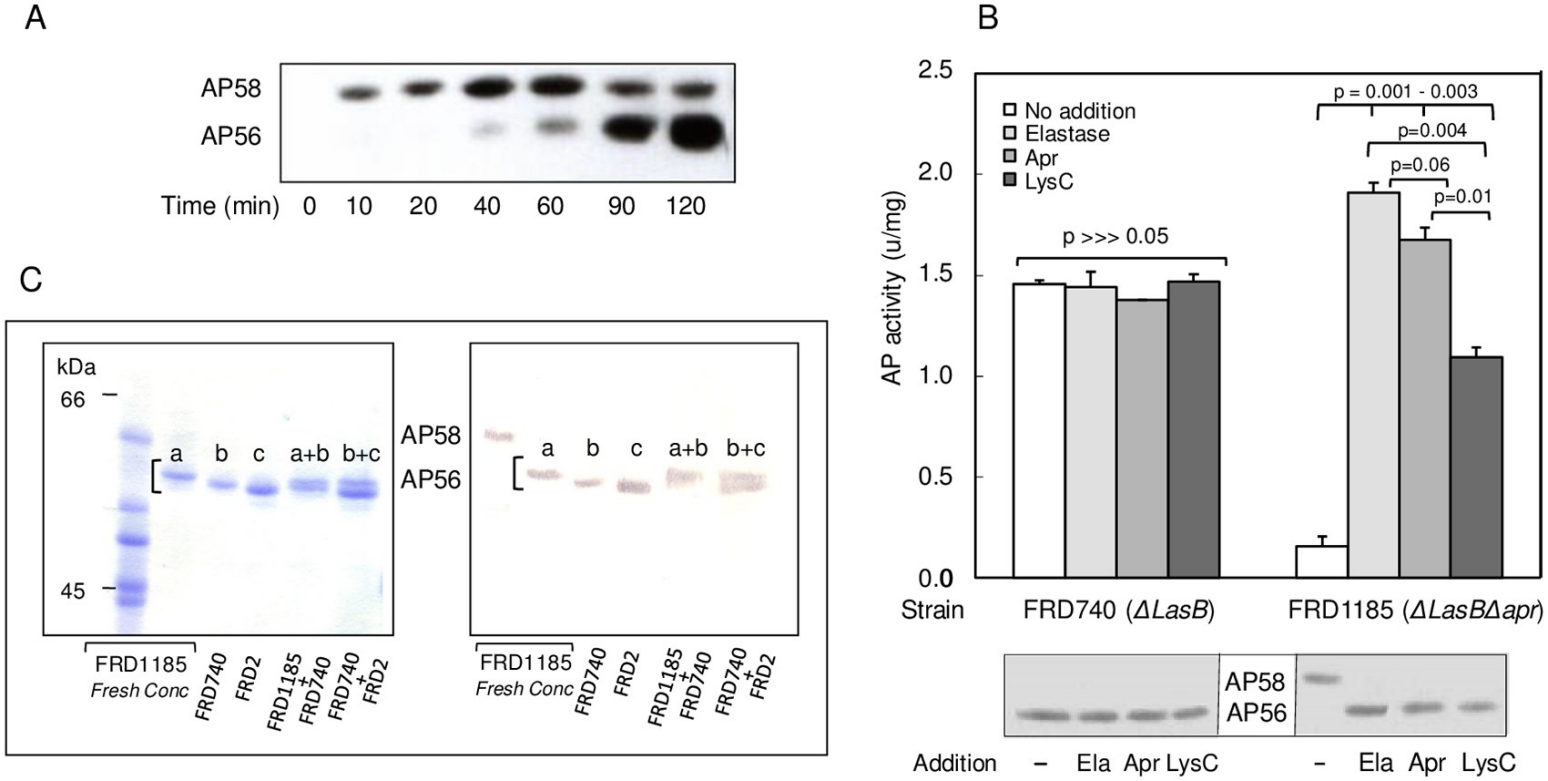
PaAP is secreted as a 58 kDa proenzyme that is processed to distinct active forms of ~56 kDa (AP56 a,b,c) each, depending on the processing protease. **A**, Progressive processing of AP58 in strain FRD2 (wild type). Bacteria grown to the late logarithmic phase were washed and incubated further in fresh medium for two hours. Samples removed at the indicated times were centrifuged and proteins in the cell-free supernatants were precipitated with trichloroacetic acid (TCA), solubilized in Laemmli’s sample buffer, and analyzed by immunoblotting with an antibody against the catalytic domain (AP28) of PaAP (see Methods). **B**, Aminopeptidase activity in culture filtrates of strains FRD740 and FRD1185 (Ela^-^Apr^+^LysC^+^ and Ela^-^Apr^-^LysC^+^, respectively; 500 μl, 0.5 μg PaAP) after incubation (1 h, 37°C) with/without addition of Ela (0.2 μg), Apr, or LysC (0.5 μg each). P values above each bar refer to differences between the columns below as indicated. **Bottom Inset**, immunoblots of the respective incubation solutions probed with a rabbit antibody to AP28. **C**, SDS-PAGE (12 cm long 8% gel, Coomassie staining, left panel) and immunoblotting (right panel) of culture filtrate samples from strains FRD1185 (Ela^-^ Apr^-^ LysC^+^, fresh and concentrated, as indicated), FRD740 (Ela^-^ Apr^+^ LysC^+^) and FRD2 (Ela^+^ Apr^+^ LysC^+^), showing that AP56 from each strain (designated AP56 a, b, c) migrates differently. FRD1185 + FRD740, mixture of culture filtrates from these strains; FRD740 + FRD2, mixture of culture filtrates from these strains. PaAP related bands in the immunoblot were detected with an alkaline-phosphatase conjugated secondary antibody (see ref. 9 for detail).

Previous studies showed that activation of pro-PaAP can be mediated by Ela [30]. Here we examined whether other secreted proteases of *P*. *aeruginosa* might also process and activate pro-PaAP. Samples of culture supernatant of *P*. *aeruginosa* FRD2 (Ela^+^ Apr^+^ LysC^+^) were collected in a time course experiment and examined by immunoblotting. This showed that PaAP emerged from the bacteria as a 58 kDa protein (AP58), accumulated in the culture medium during the first 40 min and then was gradually processed to a 56 kDa protein (AP56) (Fig 1A). This confirmed earlier predictions that PaAP is secreted as a 58 kDa pro-enzyme and is activated extracellularly by limited proteolysis [9,26,30]. When we analyzed samples of culture supernatants from *P*. *aeruginosa* strain FRD740 (Ela^-^Apr^+^LysC^+^), a mutant of FRD2 that lacked the ability to produce elastase, it still produced an enzymatically active PaAP (Fig 1B, top left). Moreover, the level of aminopeptidase activity in strain FRD740 is comparable to that found in the parental wild type strain FRD2 [9], which together with the data presented in Fig 1B (top left), indicates that Ela is not essential for activation of AP58. Addition of purified Ela did not affect PaAP activity (Fig 1B, top left). FRD740 still produced Apr and LysC, and the addition of more of these proteases also did not affect activity. Consistently, AP58 was not detected in the medium whether or not it was pre-treated with proteases, and the only PaAP related protein observed in all samples was ~56 kDa (Fig 1B, bottom left). This suggested that, in addition to Ela [30], activation of PaAP can be mediated by other proteases, such as Apr, LysC or both.

### Proteases involved in the activation of Pro-PaAP (AP58)

To test the potential role of Apr in AP58 processing and activation, an *apr*::Gm^R^ mutant of FRD740 was constructed (FRD1185), which does not produce Ela or Apr. Interestingly, when culture supernatants of FRD1185 (Ela^-^ Apr^-^LysC^+^) were tested, they were found to contain only low levels of aminopeptidase activity (Fig 1B, top right). The addition of purified Ela, Apr or LysC (endopeptidases that do not cleave the aminopeptidase substrate Leu-pNA) led to activation of PaAP (Fig 1B, top right), which showed that all three proteases can activate PaAP. However, the low level of PaAP activity in FRD1185 culture supernatant suggested that either LysC might be a minor player in pro-PaAP activation or, more likely, that in the absence of Ela and Apr, LysC was barely activated [31]. An immunoblotting analysis showed that FRD1185 produced unprocessed pro-PaAP and that conversion of AP58 to AP56 (Fig 1B, bottom right) was associated with activation (Fig 1B, top right). To further characterize AP56, culture supernatants from strains FRD2, FRD740 and FRD1185 were subjected to SDS-PAGE (Fig 1C, left panel) and immunoblotting (Fig 1C, right panel) analyses. For improved resolution, SDS-PAGE was conducted on a 12 cm long 8% polyacrylamide gel. Fresh culture supernatant from strain FRD1185 (Ela^-^ Apr^-^ LysC^+^) contained only AP58 (pro-PaAP) and not processed AP56. However, after concentration (by ultra-filtration, ammonium sulfate precipitation and dialysis), AP58 was no longer present in this medium, perhaps as a result of gradual pro-LysC activation during concentration and dialysis. FRD2 (Ela^+^ Apr^+^ LysC^+^) produced the fastest moving PaAP band (AP56c, i.e., smallest). FRD740 (Ela^-^ Apr^+^ LysC^+^) produced an AP56b of intermediate mobility. The FRD1185 (Ela^-^ Apr^-^ LysC^+^) band AP56a, which was generated during concentration and dialysis of the medium, migrated even slower. Thus, although all three proteases activated pro-PaAP, each protease appeared to process pro-PaAP differently.

To confirm the slight differences in size between the different strains, mixtures of culture supernatants from the various strains (FRD1185 + FRD740 and FRD2 + FRD740) were analyzed. As seen in Fig 1C, both mixtures exhibited doublets, suggesting that Ela, Apr and LysC each cleaved AP58 differently. The results of this experiment also supported a role for LysC in activation of pro-PaAP since AP56 was formed in the absence of Ela and Apr. This was further supported by the observation that purified LysC alone activated AP58 from strain FRD1185 *in vitro* (Fig 1B).

### Proteolytic cleavage sites and their relevance to activation of PaAP

**Fig 2:**
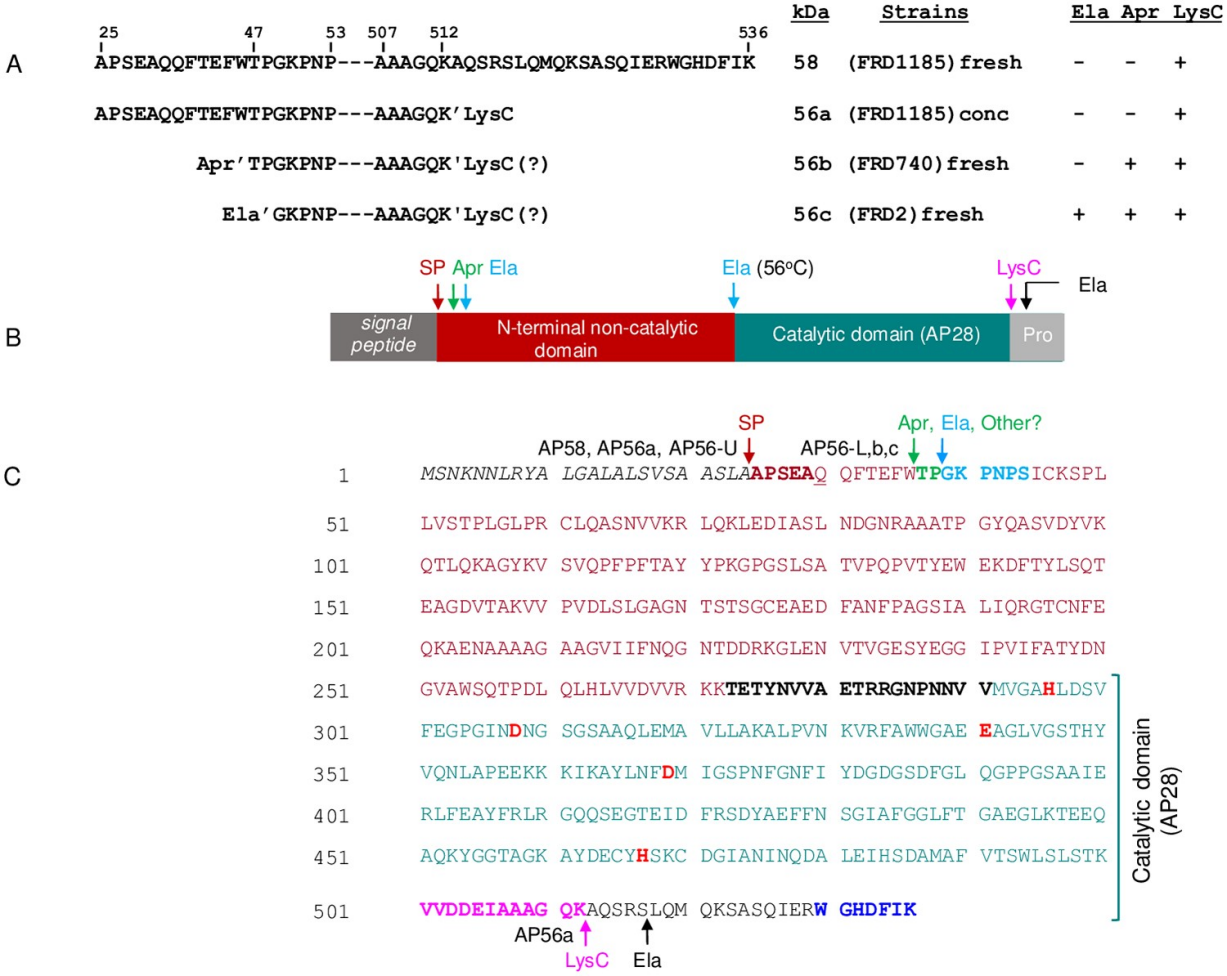
**A. Alignment of sequences showing N- and C-termini of Pro-PaAP (AP58) and PaAP variants produced by *P*. *aeruginosa* strains**. Conc indicates that Pro-PaAP in FRD1185 supernatants was concentrated, leading to C-terminal processing. Since the C-terminal sequences of AP56 b and c were not determined (see Table 1), it is not clear whether they were processed at the C-terminus by Ela or Apr, following cleavage by LysC. Hence, the C-terminal end is questionable and referred to as LysC? **B. Domain structure of pre-pro-PaAP and cleavage sites of *P*. *aeruginosa* proteases.** SP, signal peptidase; Pro, C-terminal propeptide. **C. Primary structure of pre-pro-PaAP.** Color code of each domain is the same as in **B.** The signal sequence (residues 1–24) is in *italics*. Brown arrow, cleavage site of signal peptidase (SP); green and turquoise arrows, sites cleaved by indicated proteases (Table 1) [9]; pink arrow, cleavage site of LysC (inferred from the current identification of the C-terminal sequences of AP56a, AP56U, AP56L (Table 1); black arrow, C-terminal cleavage site of Ela [30]; brown bold letters, N-terminal sequence of AP58, AP56a and AP56U (Table 1); green and turquoise bold letters, N-terminal sequences of AP56L, b and c, respectively (Table 1) [9]; black bold letters, N-terminal sequence of AP28 [9]; blue bold letters, C-terminal sequence of AP58. Red bold letters, active site residues involved in Zn-binding.

In our previous study [9], we found that the N-terminus of AP56 from the wild type *P*. *aeruginosa* strain FRD2 (GKPNP) corresponded to residues 39–43 in the pre-proenzyme (Fig 2A and 2C; Table 1, AP56c). Based on this observation, we proposed that activation resulted from the removal of the first 14 residues from the pro-enzyme. The subsequent discovery that activation of Pro-PaAP required C-terminal processing [30], in addition to our current identification of several processing variants (Fig 1C), prompted us to characterize each of these variants further to define the proteolytic events and proteases involved in physiological activation of secreted pro-PaAP. Towards this end, each processing variant was subjected to Edman degradation and Mass Spectroscopy analysis (after digestion with trypsin) to define its N- and C-terminal sequences, respectively. As was shown previously [9], the N-terminal sequence of AP56 from strain FRD740 (Ela^-^ Apr^+^ LysC^+^) was TPGKP, corresponding to residues 37–41 in pre-pro-PaAP, i.e., beginning 2 residues upstream of AP56 found in the wild type strain FRD2 (AP56c; Table 1 and Fig 2). The N-terminal sequence of AP56a, detected in concentrated medium from strain FRD1185 (Ela^-^ Apr^-^ LysC^+^) was APSEA (Table 1). The same sequence was found for AP58 and AP56 produced *in vitro* upon incubation of AP58 with LysC. The sequence APSEA corresponds to residues 25–29 in pre-pro-PaAP expected to be found after removal of the signal peptide (Table 1; Fig 2). The finding that AP56a is active even though it possesses an intact N-terminus is in agreement with the earlier findings of Sarnovsky et al [30], reinforcing their conclusion that activation results from C-terminal processing. The C-terminal sequence RWGHDFIK found for AP58 corresponds to the last eight residues of pro-PaAP (Fig 2), thus confirming that AP58 represents intact pro-PaAP. The C-terminal peptide found for AP56a, VVDDEIAAAGQK, corresponds to residues 501–512 in pre-pro-PaAP (Fig 2), indicating that activation resulted from cleavage by LysC of the peptide bond K_512_-A_513_. Fig 2A shows a linear representation of the pro-and mature PaAP variants produced by *P*. *aeruginosa* FRD strains. Fig 2B illustrates the domain structure of pre-pro-PaAP while Fig 2C shows the primary structure of pre-pro-PaAP, highlighting the individual domains as well as the specific cleavage sites and proteases involved in proteolytic processing and maturation of PaAP.

**Table 1:** N- and C-terminal amino acid sequences found for various forms of PaAP.

Strain (conditioned medium)	AP form (kDa)	Activity	N-terminus (Edman)	C-terminus (Mass Spec)
FRD2 (Ela^+^ Apr^+^ LysC^+^)	56c	+	GKPNP^1^	nd
FRD740 (Ela^-^ Apr^+^ LysC^+^)	56b	+	TPGKP	nd
FRD1185 (Ela^-^ Apr^-^ LysC^+^) fresh	58	-	APSEA	RWGHDFIK
FRD1185 (Ela^-^ Apr^-^ LysC^+^) conc	56a	+	APSEA	VVDDEIAAAGQK
AP58 + LysC	56a	+	APSEA	nd
Purified AP (upper)	56U^2^	+	APSEA	VVDDEIAAAGQK
Purified AP (lower)	56L^2^	+	TPGKP	VVDDEIAAAGQK

^1^Cahan et al [9];

^2^See Fig 5D; AP56U is identical to AP56a; AP56L is probably the same as AP56b; nd, not determined; conc, concentrated. In summary, the strain expressing only LysC produced AP56a (largest); if expressing only Apr and LysC, produced AP56b; wild type expressing Ela, Apr and LysC produced AP56c (smallest).

### LysC can directly process and activate pro-PaAP

**Fig 3:**
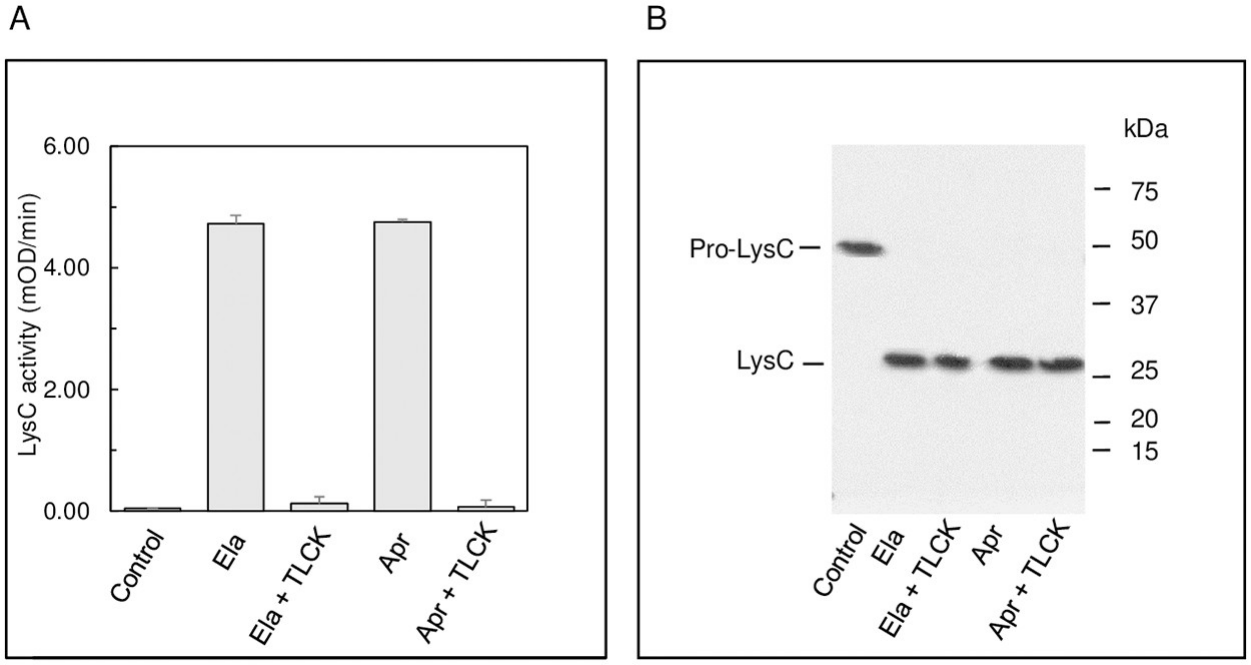
Ela and Apr can activate LysC. Culture filtrate samples from strain FRD1185 (Ela^-^ Apr^-^ LysC^+^; 150 μl) containing pro-LysC were incubated (3 h, 37°C) in the presence (+) or absence (-) of either Ela or Apr (0.45 μg each). **A**, **LysC activity**. The differences between the control (C), Ela plus TLCK and Apr plus TLCK are insignificant (p = 0.43 and 0.73, respectively); the difference between Ela and Apr is also insignificant (p = 0.78). The differences between control and Ela or control and Apr are significant (p = 0.0005 and 0.0008, respectively) and so are the differences between Ela vs Ela plus TLCK and Apr vs Apr plus TLCK (p = 0.00002 and 0.0003, respectively). **B, Immunoblot showing that both Ela and Apr can convert pro-LysC (~50 kDa) to the 28 kDa mature enzyme.** Proteins in 100 μl of each reaction solution were precipitated with TCA, solubilized in Laemmli’s sample buffer and subjected to SDS-PAGE and immunoblotting with a LysC antibody.

Exogenously added Ela and Apr can each activate pro-PaAP in the culture medium of *P*. *aeruginosa* strain FRD1185, as does LysC *in vitro* (Fig 1B). Ela has been shown to activate LysC by degrading its propeptide [32]. We wondered whether activation of pro-PaAP by Ela and Apr could be mediated by the activation of LysC, which then activates pro-PaAP by C-terminal processing. To address this question, we examined whether Ela and Apr can activate pro-LysC in the culture medium of *P*. *aeruginosa* strain FRD1185 (Ela^-^Apr^-^LysC^+^). Medium samples were incubated with either Ela or Apr, each with and without TLCK (to inhibit LysC activity), followed by determination of LysC activity. Fig 3A shows that both Ela and Apr activated LysC, and the resulting activity was inhibited by TLCK. Immunoblotting analysis showed that both enzymes converted pro-LysC (~50 kDa) to a 28 kDa protein, corresponding to mature LysC (Fig 3B). The electrophoretic pattern of LysC (Fig 3B) was the same with and without TLCK, indicating that loss of LysC activity in the presence of TLCK (Fig 3A) was solely due to inhibition and did not result from loss of the enzyme.

**Fig 4:**
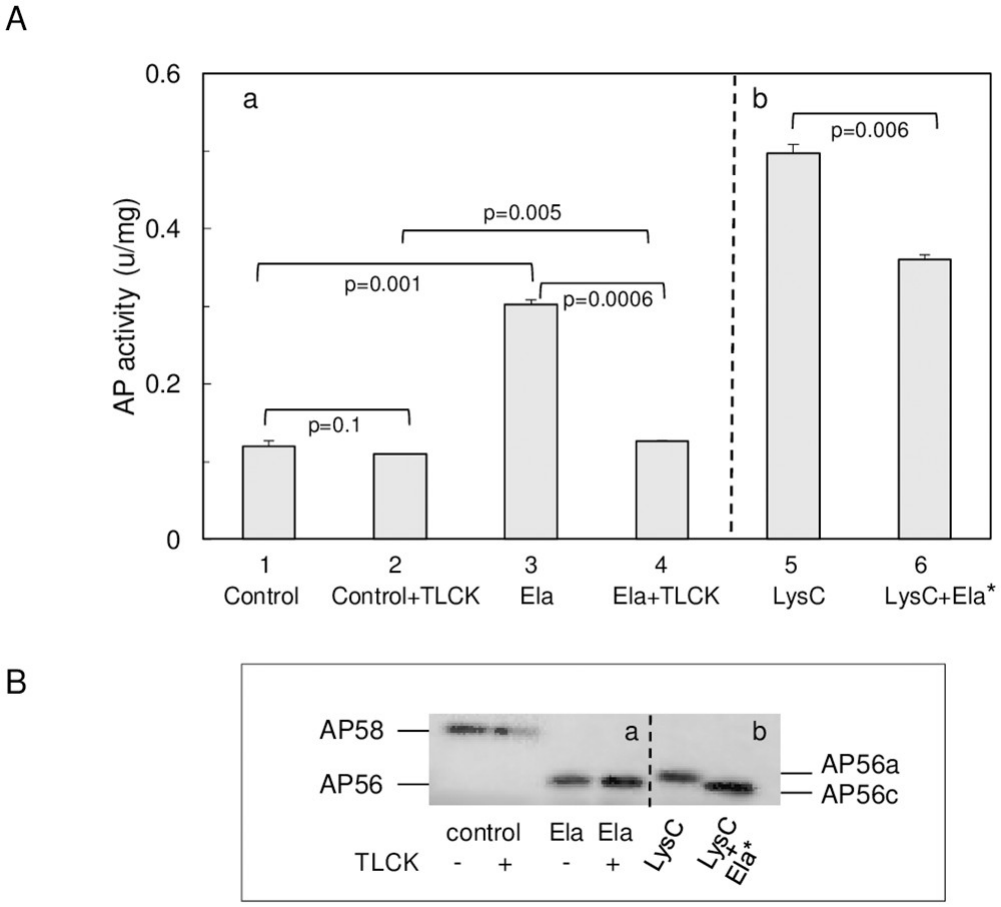
Processing and activation of pro-PaAP by Ela and LysC. **A,** (**a**), aminopeptidase activity in the culture filtrate of strain FRD1185 (Ela^-^ Apr^-^ LysC^+^) after incubation with Ela in the absence (1,3) or presence (2,4) of TLCK. Control, incubation without Ela. (**b**), aminopeptidase activity after incubation with LysC alone (5) and after addition of Ela to a sample removed from this reaction solution and incubation for an additional hour (6; LysC+Ela*). Ela and LysC activities were blocked at the end of the incubation by adding phosphoramidon and TLCK. *Ela was added after prior incubation with LysC alone. P values below each bar refer to differences between the indicated columns. The differences between the control (1) and combined LysC and Ela (5,6) are significant (p = 0.0007 and 0.009, respectively) and so is the difference between Ela and LysC treatment (3 vs 5); p = 0.003). Aminopeptidase activity (**A**) was determined fluorimetrically with Leu-7-amido-4-methyl coumarin as the substrate. **B**, Immunoblot revealing distinct migration positions (size) of active PaAP, depending on the activating protease.

To examine whether activation of pro-PaAP by Ela may be mediated by activation of LysC, we incubated medium from strain FRD1185 (Ela^-^ Apr^-^ LysC^+^) with Ela in the absence and presence of TLCK. Fig 4A (a) shows that incubation with Ela without TLCK led to activation of PaAP while no aminopeptidase activity was found in the presence of TLCK. This suggested that activation of PaAP by Ela involved two steps: activation of LysC and then C-terminal processing of pro-PaAP by LysC. Immunoblotting analysis showed that migration of the processed aminopeptidase was essentially the same whether or not TLCK was present (Fig 4B) i.e., the band corresponding to PaAP migrated faster than AP56a. This probably reflects the combined action of LysC and Ela, the former cleaving at the C-terminus while the latter processing the N-terminus. When pro-PaAP in the medium of strain FRD1185 was incubated with exogenously added LysC, aminopeptidase activity was about two-fold higher as compared to Ela alone and PaAP migrated slower (AP56a) than in the presence of Ela. Addition of Ela at this point led to the conversion of AP56a to AP56c, apparently due to N-terminal processing by Ela.

### The non-catalytic domain of PaAP has little or no effect on hydrolysis of low molecular weight substrates

**Fig 5:**
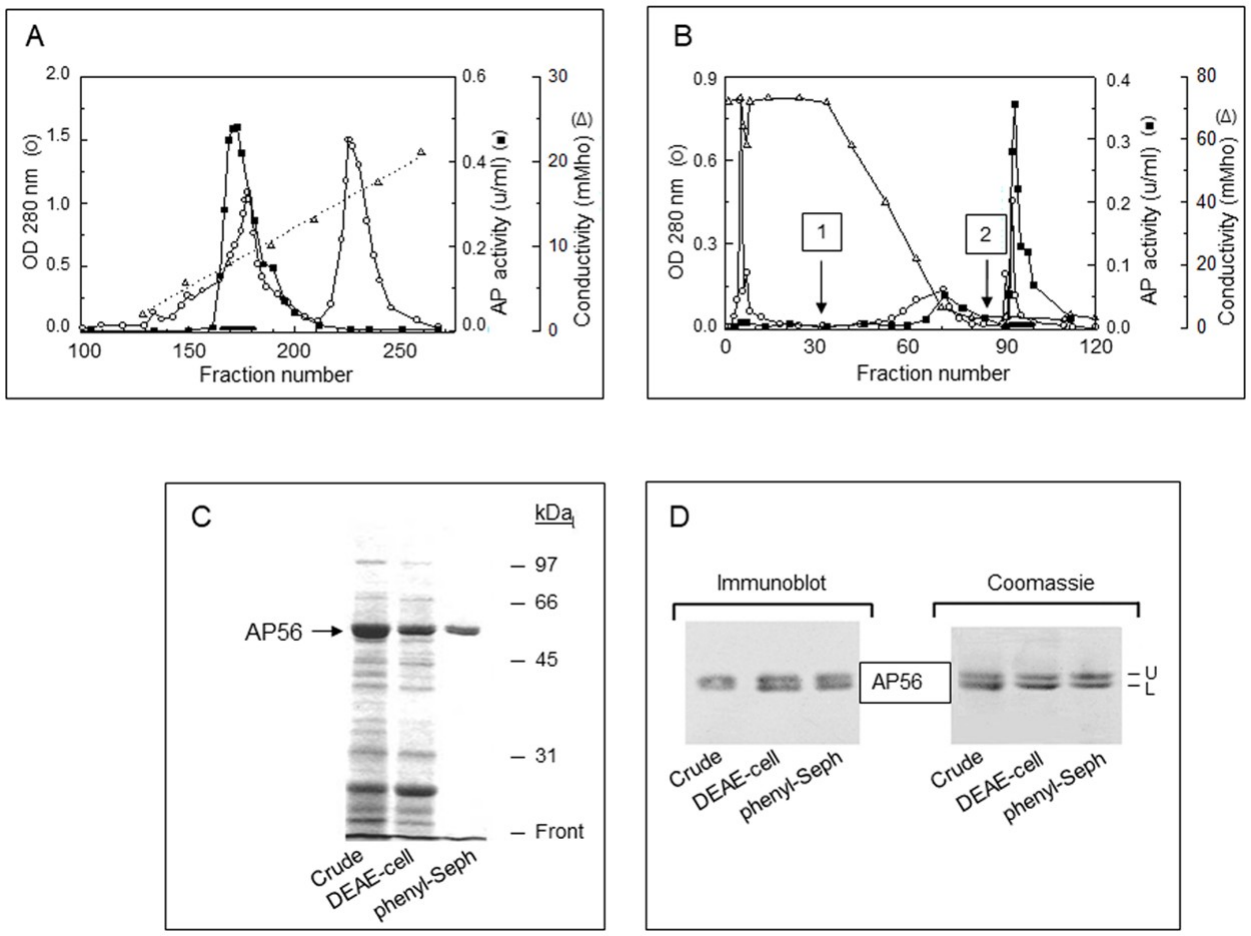
Purification of PaAP from the culture filtrate of strain FRD1185(pSS380). **A, DEAE-cellulose chromatography.** Fractions containing aminopeptidase activity (black bar) were pooled and prepared for hydrophobic chromatography as detailed in Methods. **B**, **Hydrophobic chromatography on phenyl-Sepharose.** Arrows 1 and 2, gradient elution with 0.5–0 M ammonium sulfate and step elution with 4 M urea, respectively. Aminopeptidase activity was determined after removal of the urea by dialysis. **C**, Progress of purification by SDS-PAGE (10% polyacrylamide, mini-gel, Coomassie staining). **Lanes: Crude**, concentrated culture filtrate (7 μg); **DEAE-cell**, active fraction after DEAE-cellulose chromatography (7 μg); **phenyl-Seph**, active fraction after elution from phenyl-Sepharose (0.2 μg). **D**, SDS-PAGE (long 8% polyacrylamide gel) and immunoblotting analyses showing that at all stages of purification, the enzyme fractions contained two closely migrating species of about 56 kDa. **Crude, DEAE-cell and phenyl-Seph,** the same as in panel C. Immunoblot, ~ 0.2 μg/lane; Coomassie staining, ~1 μg/lane.

The function(s) of the N-terminal non-catalytic domain retained in AP56 is not clear. To determine its potential effect on the enzyme activity, we compared the activity of AP56 (which contains the non-catalytic domain) with that of AP28 (the catalytic domain alone) towards several amino acid p-NA derivatives that are common aminopeptidase substrates. AP56 needed for these experiments was purified from strain FRD1185(pSS380) by DEAE-cellulose chromatography followed by hydrophobic chromatography on phenyl-Sepharose. Fig 5A depicts the elution pattern of the DEAE-cellulose column, showing that the bound aminopeptidase was eluted from the column at conductivity 8 mMho (~0.2M NaCl). The active fractions were pooled, concentrated, and applied onto a phenyl-Sepharose column in the presence of 0.5 M ammonium sulfate. PaAP bound strongly to the resin as it was barely released by a 0.5–0.0 M ammonium sulfate gradient. Quantitative amounts of the enzyme were only released upon elution with 4 M urea (Fig 5B). This suggested that AP56 is highly hydrophobic. The purification factor was 2.8-fold while the yield was 9.6% (Table 2). The amount of purified enzyme obtained from 1 liter of the original culture medium was 2 mg. SDS-PAGE analysis in a standard 10% polyacrylamide gel revealed a high degree of purity of AP56 (Fig 5C). Electrophoresis in a 12 cm long 8% polyacrylamide gel resolved the AP56 band into two bands migrating closely to each other as evident by both immunoblotting (Fig 5D left) and Coomassie staining (Fig 5D right). To find out whether the difference between these variants could reflect different processing reactions, we determined the N- and C-terminal sequences of each. The N-terminal sequence of the upper band (AP56U) was APSEA (Table 1), the same as that of pro-PaAP, meaning that its N-terminus was intact. Its C-terminal sequence (VVDDEIAAAGQK) was identical to that of AP56a (Table 1), implying that it was processed at the C-terminal end by LysC. The N-terminal sequence of the lower band (AP56L) was TPGKP (Table 1), indicating that it underwent N-terminal processing. Its C-terminal sequence (VVDDEIAAAGQK) was the same as that of AP56U, indicating that it was processed at the C-terminal end. Thus, the difference between the two variants lies at the N-terminus.

**Table 2:** Purification of AP56 from strain FRD1185(pSS380) Ela^-^ Apr^-^ PaAP^+++^.

Purification step	Volume (ml)	Activity (u/ml)	Protein (mg/ml)	Specific activity (u/mg)	Purification (fold)	Yield (%)
Ammonium Sulfate (80%)	30	1.0	2.1	0.5	1.0	100
DEAE-Cellulose	11	0.8	1.2	0.7	1.4	29
Phenyl-Sepharose	4.1	0.7	0.5	1.4	2.8	9.6

AP28 required for the activity assays was produced by heating AP56 in the presence of Ela [9] and the kinetic constants *K*_*m*_ and *K*_*cat*_ for hydrolysis of four amino acid p-nitroanilide derivatives by AP56 and AP28 were determined. Table 3 shows that the *K*_*m*_, *K*_*cat*_, and *K*_*cat*_/*K*_*m*_ values obtained for cleavage of Leu-, Arg- and Met-pNA by AP28 were practically the same as those derived for AP56, suggesting that the non-catalytic N-terminal domain included in AP56 has little or no effect on the rate of hydrolysis of each of these substrates. Lys-pNA behaved differently in that although the *K*_m_ values derived for AP56 and AP28 were basically the same (1.2±0.2 and 1.4±0.3 mM, respectively), the catalytic constants *K*_cat_ and *K*_*cat*_/*K*_m_ for cleavage by AP28 were ~1.7 and 1.4-fold higher than those found for AP56. This suggested that the non-catalytic domain may hamper Lys-pNA hydrolysis. Strikingly, the overall rate of cleavage of Lys-pNA by both AP56 and AP28 was 2 to 3-fold higher than that of Leu-pNA and ~8-fold faster than cleavage of Arg- and Met-pNA. Thus, out of the four amino acid pNA derivatives studied, Lys-pNA was the preferred substrate.

**Table 3:** Kinetic constants for hydrolysis of several amino acid p-nitroanilide derivatives by AP56 and AP28.

Substrate	Enzyme Form	[S] Range mM	*K*_m_ (mM)	*K*_cat_ (sec^-1^)	*K*_cat_/*K*_m_ (s^-1^x M^-1^x10^-3^)
Leu-*p*NA	AP56	0.3–4.5	7.5±1.5	18.7±3.7	2.5
	AP28		7.2±0.7	20.5±3.2	2.8
Lys-*p*NA	AP56	0.15–4.5	1.2±0.2	6.9±1.0	5.8
	AP28		1.4±0.3	11.9±1.9	8.5
Arg-*p*NA	AP56	0.3–4.8	3.9±0.7	2.7±0.6	0.7
	AP28		4.7±0.6	4.3±0.6	0.9
Met-*p*NA	AP56	0.3–4.8	4.7±0.1	3.7±1.4	0.8
	AP28		4.3±0.2	4.1±0.8	1.0

*K*_m_ and *V*_max_ values represent the mean ± SD of (at least) 4 independent measurements.

## Discussion

Similar to most secreted proteases, bacterial aminopeptidases are often secreted as inactive pro-enzymes containing inhibitory N-terminal propeptides [21,33–36]. Accordingly, in our previous study [9], we assumed that the loss of the first 12–14 residues from pro-PaAP accounted for activation. The subsequent discovery that activation of PaAP requires the removal of a short C-terminal propeptide [30] was therefore surprising. Although the evidence for this mechanism was solid, it was deduced from *in vitro* studies, using recombinant pro-PaAP as substrate and mostly non-relevant proteases as potential activators. Thus, the natural activation mechanism(s) of PaAP, addressed herein, remained obscure. The function(s) of the long N-terminal domain retained in AP56 is also poorly understood. Hence, we began studies aimed at better understanding the role of this domain, asking whether it affects enzymatic activity.

In studying processing of pro-PaAP, we focused on Ela, Apr and LysC as potential activators because they are best characterized [2,7]. LasA protease, another well-studied endopeptidase of *P*. *aeruginosa*, was excluded because PaAP is fully active in a LasA protease deficient strain [9]. In addition, there are no Gly-Gly pairs to be cleaved by LasA protease at the C-terminal region of pro-PaAP (Fig 2C).

To investigate the roles of Ela, Apr or LysC in extracellular processing of pro-PaAP, one should bear in mind the mechanisms underlying their secretion and activation. Ela and LysC are both produced as pre-pro-enzymes containing a signal peptide and an inhibitory N-terminal propeptide [31,32,37–39]. The signal peptide is removed upon passage through the inner membrane. Pro-Ela is self-processed within the periplasm, followed by rapid formation of an inactive Ela-propeptide complex that dissociates extracellularly to release activity [37,38,40]. LysC on the other hand is secreted as an inactive pro-enzyme [32,41] that is activated extracellularly [31]. Apr is secreted as a fully active enzyme whose intracellular activity is controlled by a specific inhibitor (*AprI*) rather than an inhibitory propeptide [13,25,42].

To assess the role(s) of Ela, Apr and LysC in PaAP’s activation, we followed pro-PaAP processing and aminopeptidase activity in *P*. *aeruginosa* strains lacking either Ela alone (FRD740) or Ela and Apr (FRD1185) as compared to strain FRD2 (Ela^+^ Apr^+^ LysC^+^). LysC production was not manipulated in any of these strains but since it can be activated by Ela (Fig 3) [31,32] and by Apr (Fig 3), we could dissect the role of LysC as well.

To validate that PaAP is secreted as a 58 kDa protein, we followed secretion and processing in strain FRD2 (Ela^+^ Apr^+^ LysC^+^) after washing the bacteria so that at time zero (T_o_), the medium was free of secreted proteases. The results showed clearly that PaAP emerged from the cell as a 58 kDa protein (AP58) that was processed to AP56 after ~40 min (Fig 1A), apparently, when the levels of secreted proteases in the medium were high enough. While Ela can activate pro-PaAP directly by cleaving the peptide bond Ser_517_-Leu_518_ in pro-PaAP [30], our findings support LysC as the principal activating protease. Firstly, we show that purified LysC can remove a C-terminal sequence from AP58, leading to activation (Table 1; Fig 1B; Fig 4A, part b). Moreover, AP58 is barely activated by Ela in the presence of the LysC inhibitor TLCK although it is processed to a ~56 kDa protein (Fig 4A and 4B, part a). This suggests that activation by Ela (Fig 1B) is mediated by LysC, which is consistent with the demonstration that purified LysC converts AP58 to an active species (AP56a), larger than that generated by the combined action of LysC and Ela (Fig 4B, part b). Although it is not known whether Apr can directly cleave a bond(s) within the C-terminal region of AP58, it may promote pro-PaAP activation via activation of LysC (Fig 3), which is consistent with the finding that in strain FRD740 (Ela^-^ Apr^+^ LysC^+^), secreted PaAP is active and migrates as a 56 kDa protein (Fig 1B and 1C) [9].

LysC is an extremely potent protease [15]. Thus, even in the presence of other proteases, as is the case in wild type *P*. *aeruginosa* strains, once activated, it may be the most efficient activating protease. Conversion of the active variant produced by LysC, AP56a, to AP56c (Figs 1A & 4B, part b) may reflect N-terminal processing either by Ela/Apr or by autoprocessing as was proposed by Sarnovsky et al [30]. N-terminal processing seems to be a regular step in maturation of PaAP. It was documented in strains FRD2 and FRD740 (Table 1) [9], recombinant PaAP activated *in vitro* [30] and in our purified recombinant PaAP preparation (Fig 5D, Table 1). Since N-terminal processing was prevented by mutagenesis of active site residues, Sarnovsky et al [30] proposed that it occurred autocatalytically. This proposition is puzzling because aminopeptidases do not normally cleave internal peptide bonds [10] and sequential trimming of the N-terminus by PaAP is unlikely because the N-terminal sequence of pro-PaAP comprises residues resistant to PaAP’s action such as Glu and Pro.

The demonstration that the C- and N-terminal sequences of AP58 are both intact (Table 1) verified the identity of AP58 as pro-PaAP. The first hint in the current study that activation depends on C-terminal processing of AP58 arose from the finding that AP56a is active even though its N-terminus is intact (Table 1). The sequence of the C-terminal peptide of AP56a (Table 1) indicates that activation resulted from cleavage between Lys_512_ and Ala_513_. In the absence of Ela and Apr, as is the case in strain FRD1185, and in light of the strict cleavage specificity of LysC, we propose that activation results from C-terminal processing by LysC. This is supported by the demonstration that purified LysC can activate AP58 *in vitro*. Furthermore, AP56U in the purified aminopeptidase preparation (Fig 5D) displays the same N- and C-terminal sequences as does AP56a. Interestingly, the peptide bond cleaved by LysC is also cleaved by trypsin [30], suggesting that this bond is susceptible to proteolysis and thus, likely to be the physiological processing site. While our findings point at LysC as the major activating protease of PaAP, one cannot exclude involvement of other *Pseudomonas* proteases, especially PASP and EprS, two serine proteases cleaving preferentially peptide bonds on the carboxyl side of lysine residues in proteins and peptides [43,44]. These and other *Pseudomonas* proteases may also activate LysC in strains lacking Ela and Apr such as FRD1185 and FRD1185(pSS380). Activation of LysC in these strains however is slow, perhaps because the levels of these other proteases in the culture media of *P*. *aeruginosa* are low.

Lys536, the C-terminal residue of AP58 (Fig 2) is critical for maintaining pro-PaAP inactive. Sarnovsky et al [30] proposed that inhibition is mediated by interaction of the basic side chain of Lys536 with an acidic side chain within/near the active site, thus preventing substrate access to the active site. Activation requires disruption of this interaction by proteolytic cleavage near the C-terminus. Another mechanism could be interaction of the basic side chain of Lys536 with an acidic side chain at the N-terminal end of pro-PaAP (for instance Glu4, Glu10; Fig 2). An inhibitory mechanism involving interaction between the C- and N-terminal pro-sequences was proposed for the closely related aminopeptidase of *Aeromonas proteolytica* (*Vibrio proteolyticus*) [35].

**Fig 6:**
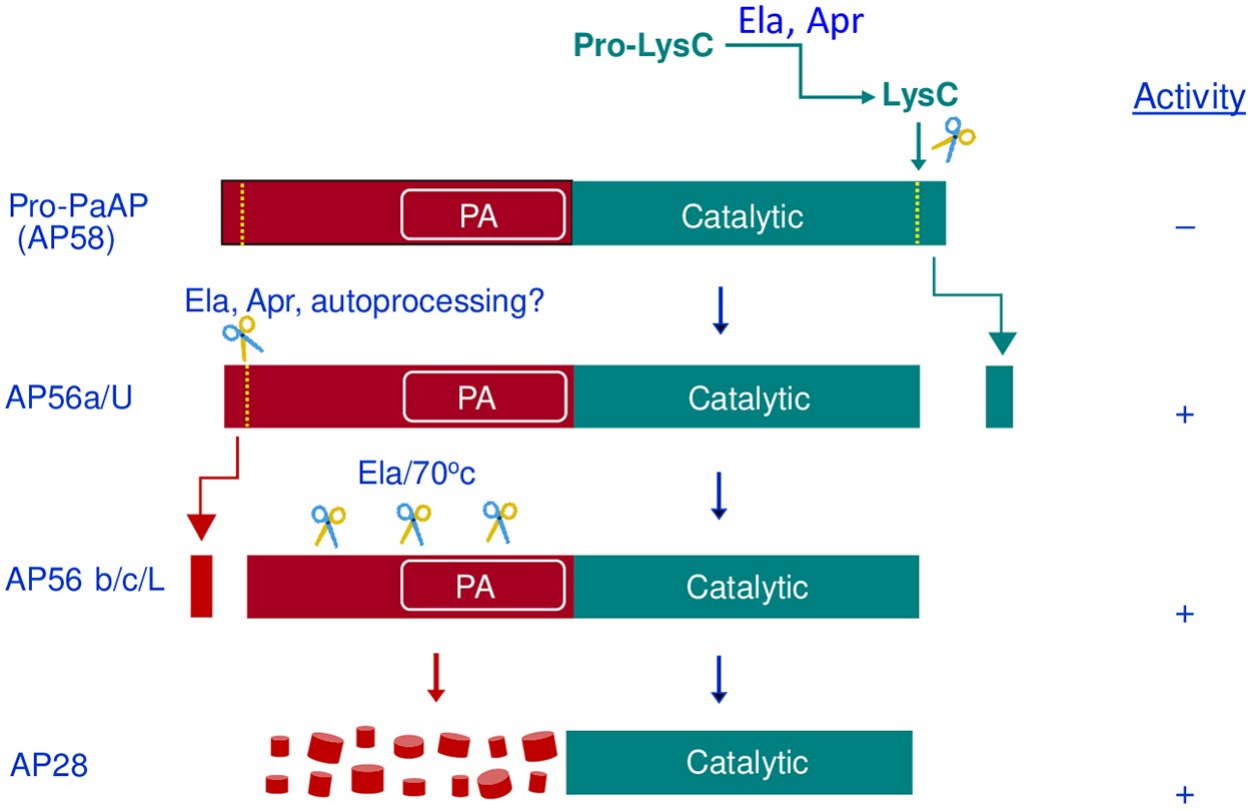
Domain structure of pro-PaAP and active forms of PaAP produced by specific *P*. *aeruginosa* proteases. **Brown**, N-terminal non-catalytic domain; **PA**, protease associated domain; **dark green**, catalytic domain. Activation results from C-terminal processing by LysC, apparently after activation by Ela and/or Apr. Cleavage by LysC generates AP56a/U; Table 1, Fig 5D). AP56a/U can be processed further at the N-terminus by Ela, Apr, or autocatalytically (or other proteases), generating AP56b/L or AP56c (Table 1; Figs 1C and 5D), apparently without effect on activity. The catalytic domain (AP28) can be released by heating of AP56 at 70°C in the presence of Ela [9]. This involves degradation of the non-catalytic domain into peptides, which may be degraded further by AP28 itself.

Fig 6 summarizes schematically the current knowledge on pro-PaAP processing and activation. Briefly, PaAP is secreted as an inactive pro-enzyme (AP58). Activation depends on cleavage of the Lys_512_-Ala_513_ peptide bond by LysC, yielding AP56a that is fully active. LysC itself must be activated first and this depends on Ela and/or Apr. In parallel, or perhaps after activation by C-terminal processing, PaAP undergoes limited N-terminal processing, either autocatalytically or by Ela/Apr. The catalytic domain of PaAP, AP28, can be released from mature PaAP artificially by heating in the presence of Ela, which apparently degrades the heat-sensitive non-catalytic domain into small fragments [9].

PaAP is found in the culture media of *P*. *aeruginosa* as a ~ 56 kDa protein, retaining a long non-catalytic N-terminal domain (Fig 6) [9] that, as in the case of the closely related *Bacillus subtilis* aminopeptidase (BSAP) [21], comprises a protease-associated (PA) domain, thought to be involved in protein-protein interactions. This is in contrast to other closely related bacterial aminopeptidases, including *V*. *cholera*, *V*. *proteolyticus*, and *A*. *caviae* aminopeptidases, that are found in the culture media as proteins of ~ 32 kDa, consisting exclusively of their catalytic domains [35,36,45]. Thus, the non-catalytic N-terminal domain of AP56 most probably fulfils a specific physiological function(s). Some possibilities include targeting to specific sites such as outer membrane vesicles (OMV) [46,47] and biofilms that are enriched in PaAP [48,49]. However, this domain may also control hydrolysis of certain substrates, a possibility we began to look at, focusing only on low molecular weight synthetic substrates.

In order to obtain a large quantity of PaAP to address our question, we overexpressed the enzyme in strain FRD1185(pSS380). Since this strain does not produce l Ela and Apr, this guaranteed processing of pro-PaAP by LysC. Just as important, the absence of Ela (the major component in culture media of most *P*. *aeruginosa* strains) simplified the purification process and resulted in PaAP being one of the major components in the medium. Consistently, the purification factor was relatively low (Table 2). As shown in Fig 5C, a highly purified enzyme preparation was obtained. The relatively low yield (9.6%) probably reflects the high hydrophobicity of the enzyme, suggested by its tight binding to phenyl-Sepharose. High-resolution SDS-PAGE and immunoblotting analyses of the purified enzyme resolved it into two processing variants (Fig 5D). The top band (AP56U) represents the product of C-terminal processing by LysC (Table 1) while the smaller variant, AP56L, is a product of AP56U, generated by N-terminal processing (Table 1). In view of the high purity of the enzyme (Fig 5C) the original suggestion that N-terminal processing could be autocatalytic [30] cannot be excluded.

Using the purified AP56, we prepared sufficient amounts of the catalytic domain (AP28), to compare the catalytic constants for hydrolysis of several substrates by both forms of the enzyme. The finding that the kinetic parameters for hydrolysis by AP28 and AP56 of all four substrates tested are comparable (Table 3) indicates that the N-terminal domain has little or no effect on hydrolysis of low molecular weight substrates, as was also shown for hydrolysis of Leu-pNA by BSAP [21]. Interestingly, deletion of the PA domain of BSAP increased the rate of hydrolysis of larger substrates dramatically [21], suggesting that this domain may restrict the access of polypeptides to the active site. The PA domain of PaAP may play a similar role.

The finding that hydrolysis of Lys-pNA by both AP28 and AP56 is 2 to 3-fold more efficient than that of Leu-pNA (Table 3) indicates that Lys-pNA is a better substrate than Leu-pNA. This is consistent with the data of Wu et al [22] who termed the enzyme lysine aminopeptidase. The *K*_*m*_ values we found for hydrolysis of Leu- and Lys-pNA by AP56 (1.2 and 7.5 mM, respectively) are also comparable to those reported by Wu et al [22], 2.32 and 9.4 mM, respectively. Because Leu-pNA is cleaved by PaAP at a reasonable rate [9], some authors [30,49] referred to it as leucine aminopeptidase. In view of its preference to N-terminal lysine residues, this terminology becomes inappropriate. Nonetheless, compared with Arg- and Met-pNA, that are cleaved 3 to 4-fold slower than Leu-pNA, it remains a useful substrate.

In conclusion, in this study, we confirmed that PaAP is secreted as an inactive proenzyme of about 58 kDa and showed that out of the many proteases secreted by *P*. *aeruginosa*, LysC is most likely the principal activating protease of PaAP, cleaving a unique peptide bond at the C-terminal end of pro-PaAP. Ela and Apr may activate PaAP indirectly via activation of LysC. In the absence of Ela and Apr, LysC may be activated slowly by minor proteases such as PASP and EprS [43,44]. We also report that PaAP shows high preference to substrates with N-terminal lysine, and that the non-catalytic domain retained in AP56 has little or no effect on hydrolysis of low molecular weight synthetic substrates. It may however play a role in targeting of PaAP to specific compartments such as OMVs [46,47] and biofilms [48,49], and perhaps, regulate hydrolysis of natural substrates.

## Materials and methods

### DNA manipulations, transformations and conjugations

For cloning, *E*. *coli* DH10B was used as the host strain. DNA was electroporated into *E*. *coli* as previously described [50] using the Gene Pulser by Bio-Rad (Hercules, CA). DNA fragments were excised and purified from agarose gels using Qiaex II DNA gel extraction kit and plasmids were purified with Qiaprep spin miniprep columns (Qiagen, Germantown, MD) according to the manufacturer’s instructions. Restriction enzymes, DNA ligase, and *Taq* were purchased from New England Biolabs (Ipswich, MA). *Pfu* DNA polymerase was purchased from Stratagene (La Jolla, CA). All enzymes were used according to the manufacturers’ instructions. Conjugation via triparental mating to introduce plasmid DNA into *P*. *aeruginosa* was performed as previously described [50]. *Pseudomonas* Isolation Agar from Difco was used to counter select against *E*. *coli* during triparental mating. An alkaline protease (*apr*) mutant of FRD740 (Δ*lasB*) was constructed by cloning the *apr* gene by PCR, inserting a gentamycin-resistance cassette (Gm^R^) into the *apr* open reading frame, and crossing it into the FRD740 chromosome for allelic replacement to form strain FRD1185.

### Construction of *P*. *aeruginosa PepB* overproducing plasmid

The gene encoding PaAP (*pepB*) was PCR amplified from *P*. *aeruginosa* FRD1 as a 1645 bp *Nco*I-*Hin*dIII DNA fragment and cloned into the *E*. *coli*-*P*. *aeruginosa* shuttle and expression vector pMF54 under the transcriptional and translational control of P_*trc*_ and the ribosome binding site (RBS) of *lac*. Briefly, the DNA fragment carrying the *pepB* ORF was amplified from FRD1 using *Pfu* DNA polymerase to minimize errors during the amplification and SSO-175 (F: 5’- AAGGAACGGAGTC**C**CATG**G**GCAACAAGAAC-3’) and SSO-176 (R: 5’-CATAAGCTTCCGGCGCAGGGTAGTCGCGG-3’) as primers. The engineered *Nco*I site in SSO-175 is underlined and two nucleotides that were mutagenized to create the site for in-frame cloning into pMF54 are denoted in bold. The nucleotide-change of A to G at position +4 of the ORF to create the *Nco*I site resulted in changing of the second amino acid of the signal peptide of pre-pro-PaAP from serine (AGC) to glycine (GGC). The engineered *Hin*dIII site in SSO-176 to facilitate cloning is underlined. The primers were annealed at 64°C and the DNA was polymerized for 3.5 minutes. The 1645 *Nco*I-*Hin*dIII fragment was first digested with *Hin*dIII and then partially digested with *Nco*I. The partial digest was necessary because *pepB* carries a unique *Nco*I site approximately 150 nucleotides upstream from the 3’-end of the ORF. The 1611 *Nco*I-*Hin*dIII fragment was gel-purified and ligated into the *Nco*I-*Hin*dIII digested pMF54 at 16°C overnight. The ligation mixture was digested with *Kpn*I to linearize vector plasmids lacking the insert to minimize isolation of plasmids lacking the insert, electroporated into DH10B, and then Ap^R^ resistant colonies were selected. The clones carrying the *pepB* gene were verified via PCR and one particular clone, pSS380, was verified via DNA sequencing of the insert. The insert DNA carried on pSS380 was sequenced by the ACGT, Inc. (Wheeling, IL) and the sequence was analyzed with the Lasergene software (DNA Star, Madison, WI).

### Bacterial strains, media and growth conditions

Strains and plasmids used in this study are listed in Table 4. *E*. *coli* DH10B was grown in L broth (LB) or LB supplemented with 100 μg/ml of ampicillin. *P*. *aeruginosa* was grown in LB or LB supplemented with 100 μg/ml of carbenicillin when it carried the plasmids pMF54 or pSS380. For studies on PaAP processing and activation, *P*. *aeruginosa* strains FRD2, FRD740 or FRD1185 were grown in tryptic soy broth without glucose (Difco). For large-scale production of recombinant PaAP, *P*. *aeruginosa* strain FRD1185(pSS380) was grown in modified M9 medium [51] containing 100 μg/ml carbenicillin. Enzyme production was induced at 8 h by adding IPTG (1 mM; Sigma-Aldrich) and incubation was continued for 10 additional hours. All of the strains were grown at 37°C with aeration, unless otherwise indicated. Growth medium was solidified with 1.5% of Bacto Agar (Difco, Franklin Lakes, NJ). Antibiotics were purchased from Sigma-Aldrich, Inc. (St. Louis, MO).

**Table 4:** Bacterial strains and plasmids used in this study.

Strain or plasmid	Genotype or relevant characteristic	Source or reference
**Plasmids**		
pKK233-2	P*trc* Ap/Cb^R^	Lab collection
pMF54	pKK233-2 *oriT* SF *lacI*^*q*^	[52]
pSS380	pMF54 P*trc>pepB* (PaAP^+++^)	This study
***E*. *coli***		
DH10B	F^-^ *mcrA* Δ(*mrr-hsdRMS-mcrBC*) ϕ*80dlacZ*Δ*M15* Δ*lacX74 deoR recA1 endA1 araD139* Δ(*ara*, *leu*)*7697 galU galK l-rpsL nupG*	Thermo Fisher
DH10B(pMF54)	Ap^R^	Lab collection
DH10B(pSS380)	Ap^R^ PaAP+	This study
***P*. *aeruginosa***		
FRD1	*mucA22* Alg^+^ CF isolate	[53]
FRD2	Spontaneous Alg^-^ derivative of FRD1	[54]
FRD740	FRD2 Δ*lasB*/Tn*501* Hg^R^ (Ela^–^)	[39]
FRD1185	FRD2 Δ*lasB*/Tn*501 apr*::Gm^R^ (Ela^−^Apr^−^)	This study
FRD1185(pMF54)	Cb^R^	This study
FRD1185(pSS380)	Cb^R^ PaAP^+++^	This study

### Preparation of cell-free culture filtrates for studies on PaAP processing and activation

*P*. *aeruginosa* strains FRD2, FRD740 and FRD1185 were grown in tryptic soy broth without glucose for 24 h. Bacteria were removed by centrifugation and the clear supernatants were stored in aliquots at -20°C until use, usually up to 3 days. In an experiment designed to follow extracellular pro-PaAP processing, FRD2 bacteria were harvested at the late logarithmic phase of growth (OD at 660 nm of ~2) by centrifugation (6,000 g; 20 min, 4°C). Pelleted bacteria were washed with sterile medium, re-suspended in the original volume of fresh medium and incubated at 37°C for different time intervals. At each time point, a sample removed from the culture suspension was centrifuged immediately (Beckman microfuge; 1 min; room temperature) to remove bacterial cells. The supernatants thus obtained were re-centrifuged (4 min), supplemented with bovine serum albumin (100 μg; carrier) and proteins in the supernatants were precipitated with trichloroacetic acid (TCA). After washing, the pellets were solubilized and prepared for SDS-PAGE as described [41].

### Purification of recombinant PaAP

Cells from 2-liter cultures of *P*. *aeruginosa* FRD1185(pSS380), grown in modified M9 medium as above, were removed by centrifugation and proteins in the supernatants were concentrated by ammonium sulfate precipitation (80% saturation) as previously described [9]. Thirty ml of the concentrated culture filtrate (about 10 mg PaAP) were dialyzed against 0.02 M Tris-HCl, 0.5 mM CaCl_2_, pH 7.5 and applied on a Diethylaminoethyl (DEAE)-cellulose (DE52, Whatman) column (2.1x26 cm), equilibrated and washed with the same buffer (4°C; 20 ml/h). Bound aminopeptidase was eluted from the column with a linear NaCl gradient (0–0.6 M; 540 ml), collecting 3 ml fractions. Protein distribution was determined by measuring absorbance at 280 nm and aminopeptidase activity distribution was analyzed using a spectrophotometric assay, with Leu-pNA as the substrate (see below). Fractions containing aminopeptidase activity were pooled and concentrated to ~3 ml by ultrafiltration (membrane YM-10, Amicon, 10,000 Da cutoff). Ammonium sulfate was added to a final concentration of 0.5 M followed by dialysis against 0.05 M Tris-HCl, 0.5 mM CaCl_2_, pH 7.5 (buffer B) containing 0.5 M ammonium sulfate. The dialyzed sample containing 1 mg of PaAP was loaded on a 1 ml Hi Trap Phenyl-Sepharose column (Amersham; currently Cytiva) pre-equilibrated with the same buffer and chromatographed using an ACTA FPLC instrument (Amersham; currently Cytiva) at 4°C, collecting 0.5 ml fractions. After washing with equilibration buffer (10 ml), the column was washed with an ammonium sulfate linear gradient of 0.5–0.0 M (20 ml). Almost all of the aminopeptidase activity remained adsorbed to the column and was eluted with 4 M urea in buffer B. Aminopeptidase activity was determined following dialysis of representative fractions against buffer B, and the active fractions were pooled, concentrated with Aquacide, dialyzed again against buffer B and stored in aliquots at -80°C.

### *P*. *aeruginosa* proteases

Ela was purified as previously described [55]. LysC endopeptidase was obtained by DEAE-cellulose chromatography at pH 8.3 of culture filtrate from strain FRD740 [9] where it was found in the run-through fractions. Fractions containing LysC were pooled, concentrated and applied to a carboxymethyl (CMC)-cellulose column equilibrated with 0.02 M phosphate buffer pH 6.0. LysC adsorbed to the column and was eluted with a linear gradient of 0–0.6 M NaCl in the same buffer. Apr was purified by DEAE-cellulose chromatography at pH 7.5 performed as previously described [9]. Apr adsorbed to the column and was eluted using a linear gradient of 0–0.6 M NaCl in equilibration buffer (0.02 M Tris-HCl, 0.5 mM CaCl_2_, pH 7.5). Purified LysC and Apr (both homogenous as judged by SDS-PAGE and Coomassie blue staining) were dialyzed against 0.05 M Tris-HCl, 0.5 mM CaCl_2_, pH 7.5 and stored in aliquots at -80°C.

### Enzyme assays

Aminopeptidase activity was determined either spectrophotometrically with Leu-p-nitroanilide (Leu-pNA; Sigma) or fluorometrically with Leu-7-amido-4-methyl coumarin (Leu-AMC; Sigma) as substrates, respectively. Leu-pNA (0.6 mM in 100 μl 0.05 M Tris-HCl pH 8.5) was incubated (30°C) with the enzyme in a 96-wells plate and enzyme activity was monitored by measuring the rate of increase in absorbance at 405 nm (due to release of p-nitroaniline) using a spectrophotometric ELISA plate reader (Biotek, ELX808). In the fluorometric assay, reactions were conducted similarly using 96 dark multi-well plates (Greiner). Each well contained 100 μl of 0.22 mM Leu-MCA in 0.05 M Tris-HCl pH 8.5 and the rate of increase in fluorescence (due to release of free MCA by the enzyme) was monitored using an ELISA plate fluorimeter (Bio-Tek FLx 800) with excitation at 360 nm and emission at 460 nm. Activity was expressed as u/mg. One unit of activity is the amount of enzyme that releases 1 μmole p-nitroaniline or 1 μmole 7-amino-4-methyl-coumarin, respectively. LysC activity was determined in a 96 well plate using Tosyl-Gly-Pro-Lys-p-nitroanilide (Chromozym PL; Sigma-Aldrich) as the substrate. 100 μl of 0.02 M Tris-HCl, 1 mM EDTA, pH 8.0 containing 0.2 mM substrate and an appropriate enzyme aliquot were incubated at 30°C, and the rate of cleavage was determined by monitoring the rate of increase of absorbance at 405 nm due to the release of p-nitroaniline using an ELISA plate reader (Biotek, ELX808) (See ref. 8 for further details on all of the above procedures).

### Kinetic constants

Kinetic constants *K*_*m*_ and *K*_*cat*_ for hydrolysis of p-nitroanilide (pNA) derivatives of leucine, methionine, lysine and arginine by AP56 and AP28 were determined using the spectrophotometric assay. Substrate concentrations for Leu-pNA hydrolysis were in the range 0.6–4.8 mM and 0.5–4.0 mM for AP28 and AP56, respectively. Lys-pNA hydrolysis by AP28 and AP56 was studied at the concentration range of 0.15–2.4 mM. Hydrolysis of Arg- and Met-pNA by both AP28 and AP56 was studied at concentrations 0.3–4.8 mM. The enzyme concentration used for Leu- and Lys-pNA hydrolysis was 5.4 x 10^−8^ M (1.5 and 3 μg/ml for AP28 and AP56, respectively) while Arg- and Met-pNA hydrolysis was studied at an enzyme concentration of 1.3x10^-7^ M (3.7 and 7.5 μg/ml, respectively). Molar concentration of AP56 was calculated using the theoretical molecular mass value of 55 kDa [30]. *K*_*m*_ and *V*_*max*_ values were derived from Lineweaver–Burk plots.

### Antibodies

Antibodies against the catalytic domain of PaAP (AP28) were raised in rabbits as described [9]. Specific antibodies were isolated by adsorption to AP56 (~50 μg) after separation by SDS-PAGE and transfer to nitrocellulose membrane. The protein band corresponding to AP56 was localized by staining (0.1% Ponceau S in 7% TCA), excised and washed with TBS (Tris buffered saline). The membrane was then blocked (1% BSA in TBS+0.05% Tween 20) and incubated (16 h, 4°C) with the immune serum (30 ml; diluted 1:100 in blocking buffer, equivalent of 300 μl original immune serum). The membrane was then washed extensively with TBS+Tween 20, followed by elution of the antibodies with 5 mM Glycine-HCl, 0.01% BSA, 0.5 M NaCl, pH 2.3 (10 min with shaking). The pH was adjusted to 7.0 with 1 M Na_2_HPO_4_ and BSA was added to a final concentration of 0.1%. The purified antibody fraction was stored in aliquots at -80°C. Antibodies against LysC were raised in rabbit by sub-cutaneous injection of gel-pieces containing LysC after separation by SDS-PAGE using a previously described [9] procedure. Approximately 50 μg LysC were administered at the first injection, followed by 5 additional injections (20–30 μg LysC each) at 10-days intervals. Rabbits were bled 10 days after the last injection. Antibodies were purified from the immune serum by adsorption to LysC immobilized on a nitrocellulose membrane after SDS-PAGE separation as outlined above for isolation of PaAP specific antibodies.

### SDS-PAGE and immunoblotting

SDS-PAGE was performed with 4% stacking gels and either 8% or 10% polyacrylamide separating gels, as specified. Protein bands were detected by Coomassie staining. Electrophoretic transfer of proteins to nitrocellulose was performed either in 38.4 mM glycine, 25 mM ethanolamine, 20% methanol, 0.01% SDS, pH 9.4 (1.5 mm gels; Protean II system, Bio-Rad; 12 cm long separation gel) or 25 mM Tris-HCl, 192 mM glycine, 20% methanol, pH 8.3 (0.75 mm gels; Mini Protean II system; Bio-Rad). Membranes were blocked with 3% skim milk in TBS + 0.05% sodium azide followed by incubation with specific rabbit antibody in blocking buffer, washing with 0.05% Tween 20 in TBS and incubation with horse radish peroxidase-conjugated (goat) anti-rabbit IgG, followed by washing and enhanced chemiluminescence (ECL) detection. As an exception, in Fig 1C, PaAP-related bands were visualized with alkaline phosphatase-conjugated goat anti rabbit IgG (see ref. 9 for detail).

### N-terminal sequence analysis

PaAP samples from strains FRD2, FRD740 and FRD1185 (~ 50 pmoles each) were separated by SDS-PAGE and electrotransferred to polyvinylidene difluoride membranes (PVDF; Immobilon-P; Millipore Corp.) as described [15]. After staining with Coomassie Blue, protein bands corresponding to each of the PaAP forms were excised and their N-terminal sequences were determined by automated Edman degradation on an Applied Biosystems 490 protein sequencing system.

### Mass spectra analyses

Proteins were separated by SDS-PAGE (8% polyacrylamide gels; Bio-rad, Protean II system), fixed in 50% ethanol, 12% acetic acid and stained in 0.08% Coomassie Brilliant Blue G-250, 20% ethanol, 8% ammonium sulfate, 0.8% phosphoric acid. Gels were de-stained using 20% ethanol and washed in water. Protein bands of interest were excised, fragmented with trypsin and peptide mixtures were electro-sprayed into an electrospray ion trap mass spectrometer (LCQ, Finnigan, San-Jose, CA). Mass spectrometry analysis was performed in Prof. Admon’s laboratory, Technion-Israel Institute of Technology, Haifa, Israel [56].

### Statistical analyses

Data are presented as means ± SD. Statistical analysis was performed using two tail unpaired t-tests. Differences between groups were considered significant for p values <0.05.

## Supporting information

S1 File Raw image Fig 1. (PDF)

S2 File Raw image Fig 3B. (PDF)

S3 File Raw image Fig 4B. (PDF)

S4 File Raw image Fig 5. (PDF)
