# Supplementary material for: Extracellular proteolytic activation of Pseudomonas aeruginosa aminopeptidase (PaAP) and insight into the role of its non-catalytic N-terminal domain
Source: PLoS One. 2021 Jun 16;16(6):e0252970. doi: 10.1371/journal.pone.0252970 (PMC8208579; doi:10.1371/journal.pone.0252970)
Supplement: S1 File — (PDF) [file pone.0252970.s001.pdf]

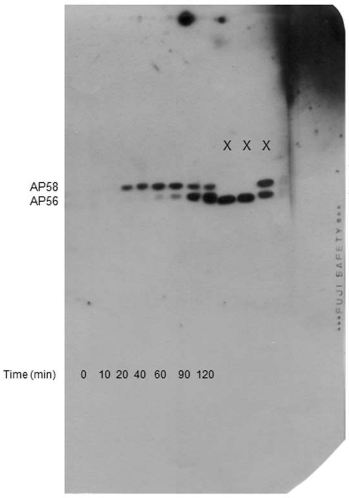

Figure 1A
12 cm long 8% acrylamide gel
Immunoblot ECL detection

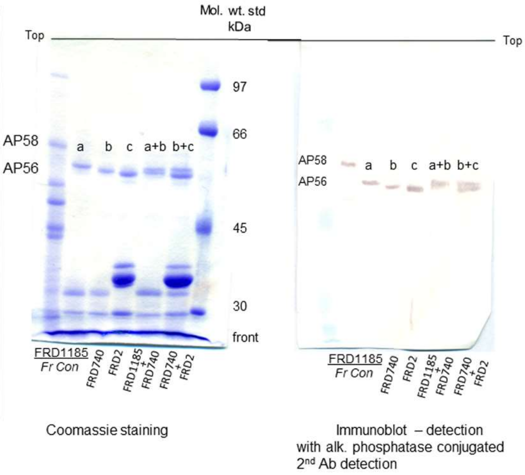

Figure 1 C
12 cm long 8% acrylamide gel

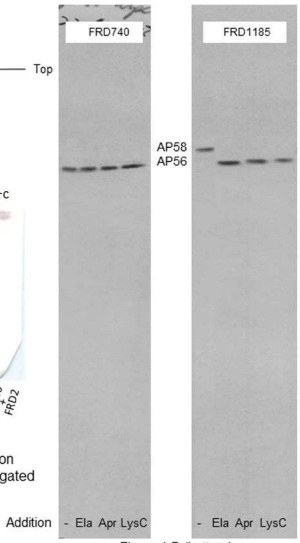

Figure 1 B (bottom)
12 cm long 8% acrylamide gel
ECL detection
