# Supplementary material for: Extracellular proteolytic activation of Pseudomonas aeruginosa aminopeptidase (PaAP) and insight into the role of its non-catalytic N-terminal domain
Source: PLoS One. 2021 Jun 16;16(6):e0252970. doi: 10.1371/journal.pone.0252970 (PMC8208579; doi:10.1371/journal.pone.0252970)
Supplement: S2 File — (PDF) [file pone.0252970.s002.pdf]

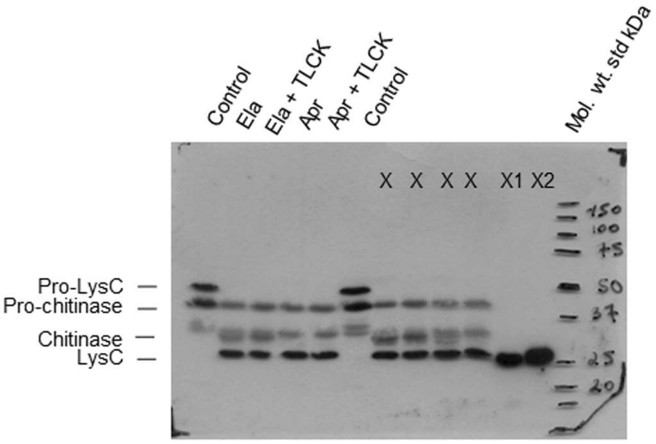

Fig 3B

(Immunoblot with an antibody to LysC)
10% minigel, scan

The anti lysC serum reacted with another extracellular product of *P. aeruginosa*, presyumably, the secreted chtinase. For clarity, the respective (non-specific) bands were removed from Fig. 3B in the manuscript. X1 and X2, standard purified LysC.
