# Supplementary figures and images for: Extracellular proteolytic activation of Pseudomonas aeruginosa aminopeptidase (PaAP) and insight into the role of its non-catalytic N-terminal domain
Source: PLoS One. 2021 Jun 16;16(6):e0252970. doi: 10.1371/journal.pone.0252970 (PMC8208579; doi:10.1371/journal.pone.0252970)

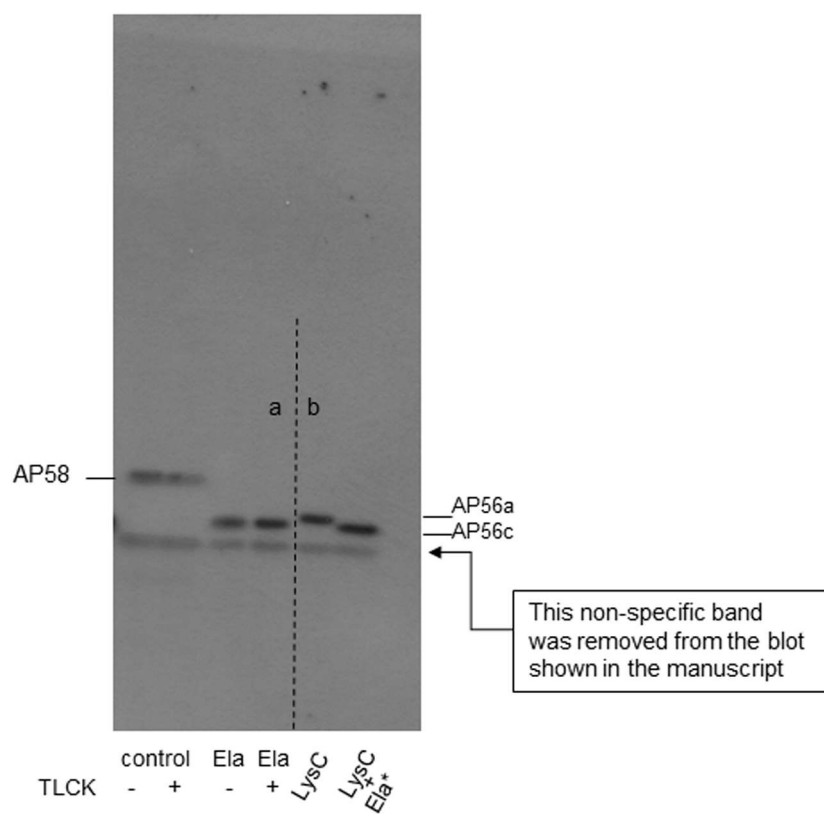

Figure 4B  
12 cm long 8% gel

Supplement: S3 File — (PDF) [file pone.0252970.s003.pdf]

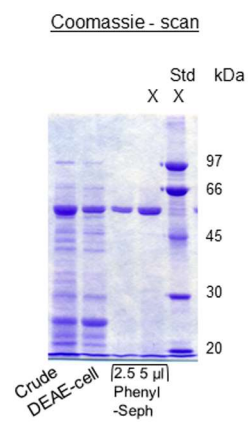

**Figure 5 C**  
(minigel 10%)

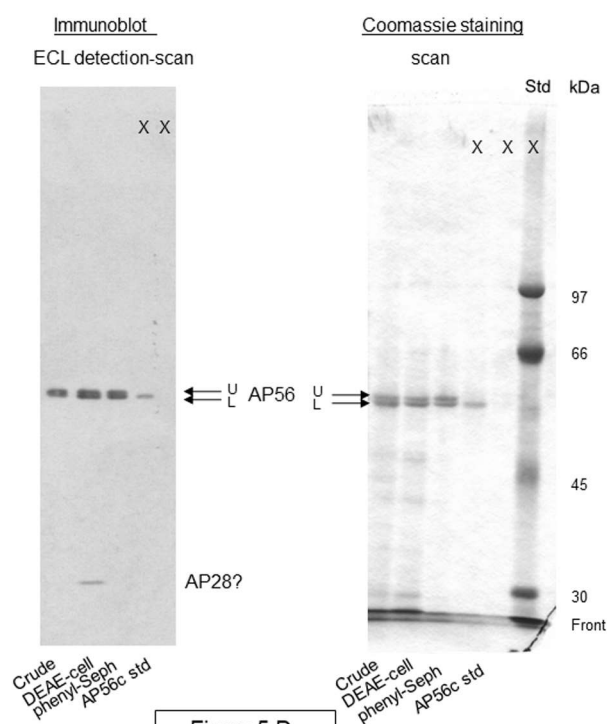

**Figure 5 D**  
(12 cm long 8% gel)

Supplement: S4 File — (PDF) [file pone.0252970.s004.pdf]
